# The Effects of the Harmful Algal Bloom Species *Karenia brevis* on Survival of Red Porgy (*Pagrus pagrus*) Larvae

**DOI:** 10.3390/toxins14070439

**Published:** 2022-06-28

**Authors:** Richard Wayne Litaker, Alex K. Bogdanoff, Donnie Ransom Hardison, William C. Holland, Andrew Ostrowski, James A. Morris

**Affiliations:** 1 CSS Inc. Under Contract to National Oceanic and Atmospheric Administration, National Ocean Service, National Centers for Coastal Ocean Science, Beaufort Laboratory, Beaufort, NC 28516, USA; 2 JHT Under Contract to National Oceanic and Atmospheric Administration, National Ocean Service, National Centers for Coastal Ocean Science, Beaufort Laboratory, Beaufort, NC 28516, USA; abogdanoff585@gmail.com; 3 The Department of General Education, James Sprunt Community College, Kenansville, NC 28349, USA; 4 National Oceanic and Atmospheric Administration, National Ocean Service, National Centers for Coastal Ocean Science, Beaufort Laboratory, Beaufort, NC 28516, USA; rance.hardison@noaa.gov (D.R.H.); chris.holland@noaa.gov (W.C.H.); james.morris@noaa.gov (J.A.M.); 5 National Oceanic and Atmospheric Administration, National Marine Fisheries Service, Southeast Fisheries Science Center, Beaufort Laboratory, Beaufort, NC 28516, USA; andy.ostrowski@noaa.gov

**Keywords:** brevetoxins (BTxs), effective concentration sufficient to kill 50% of the larvae (EC_50_), fish, Gulf of Mexico, harmful algal blooms (HABs), recruitment

## Abstract

The harmful algal bloom species, *Karenia brevis*, forms annual, often intense blooms in the Gulf of Mexico, particularly along the west Florida shelf. Though the ability of *K. brevis* blooms to cause mass mortalities in juvenile fish are well documented, the direct effect of bloom concentrations on larval fish has not been studied extensively. To better understand the potential effect of *K. brevis* on larval fish survival, laboratory spawned red porgy (*Pagrus pagrus*) larvae from 4–26 days post-hatch were exposed to concentrations of *K. brevis* observed in the field for either 24 or 48 h. This species is representative of fish which spawn in regions of the Gulf of Mexico and whose larvae are epipelagic and may encounter *K. brevis* blooms. In this study, three different *K. brevis* strains varying in the amount of brevetoxin produced were tested. Larval survivorship was found to be inversely proportional to the amount of brevetoxin produced by each strain. The EC_50_ value from the combined 24 h experiments was ~163,000 *K. brevis* cells L^−^^1^, which corresponds to cell concentrations found in moderately dense blooms. Larval mortality also increased substantially in the 48 h versus 24 h exposure treatments. These findings indicate *K. brevis* blooms have the potential to contribute to natural mortality of fish larvae and further reduce inter-annual recruitment of fishery species whose stocks in the Gulf of Mexico may already be depleted.

## 1. Introduction

The harmful algal bloom species, *Karenia brevis*, forms annual and often intense blooms throughout the Gulf of Mexico [1]. This toxic dinoflagellate species produces brevetoxins (BTx) which are potent lipophilic polyether neurotoxins that disrupt nerve function in vertebrates by activating voltage-gated sodium channels [2,3]. Though the majority (~90%) of the BTxs are retained within the cells during a bloom [4], they can nonetheless rapidly permeate the food chain. This occurs primarily when filter-feeding bivalves, deposit feeders, copepods, and planktivorous fish inadvertently consume *K. brevis* cells and rapidly bioaccumulate the lipophilic BTxs as cells are digested [5,6,7,8]. As these organisms are consumed by higher trophic level predators, the incorporated BTxs rapidly vector through the food chain [7]. BTxs are also released into the water column when cells senesce or are lysed by physical forces such as wave action. These released toxins readily absorb on numerous surfaces including seagrasses, macroalgae and detrital materials [9,10]. Consumption of these passively contaminated food sources allows BTxs to further move through the food chain where they can cause sublethal effects and direct mortalities of many organisms including zooplankton [11,12], fish [13], seabirds [14], dolphins [15,16], and manatees [17,18]. Due to the high toxicity of BTxs and multiple routes of exposure, toxic *K. brevis* blooms have been associated with massive fish kills and high mortalities of seabirds, turtles, blue crabs, jellyfish, dolphins, and manatees off the west coast of Florida dating back to 1844 [17,19,20,21,22]. BTxs released from cells can also become aerosolized and carried onshore by winds where they cause significant respiratory illness and increased hospitalizations in affected communities [2,23,24,25,26].

Interestingly, studies using isolated BTxs have shown they are highly toxic to fish, birds and mammals, but less so to many adult shellfish species [20]. Though shellfish larvae may be susceptible to high *K. brevis* concentrations [27], adults appear largely resistant to BTx toxicity. This accounts for why adult shellfish can accumulate sufficient BTx concentrations to cause Neurotoxic Shellfish Poisoning when consumed and is the primary reason why *K. brevis* cell concentrations and shellfish toxicity are so closely monitored in Florida [19,28].

While the negative effects of *K. brevis* on juvenile and adult fish have been well documented, their effects on larval fish are based on qualitative field studies and limited experimental trials. Warlen et al. [29] reported significant changes in larval fish recruitment in Onslow Bay during the 1987 North Carolina *K. brevis* bloom and Flaherty and Landsberg [30] reported significant changes in the nekton community structure in Tampa Bay during the 2005 Florida red tide. While these studies show significant population and community level changes during *K. brevis* blooms, they do not explicitly address the extent to which *K. brevis* can adversely affect fish larvae.

To date only two studies have directly examined the effects of *K. brevis* on larval fish, and neither estimated EC_50_ values. In the first study, Riley et al. [31] exposed red drum (*Sciaenops ocellatus*) eggs, yolk-sac larvae, and 21 days post hatch (dph) larvae to *K. brevis* collected during the 1986 *K. brevis* bloom in Port Aransas, Texas. They showed that exposure to *K. brevis* did not affect egg survival or hatch success, but did cause severe developmental abnormalities (e.g., spinal curvature) and high mortality in the hatched yolk-sac larvae. They also noted the 21 dph larvae needed longer exposure times to reach the same level of mortality as the yolk-sac larvae [31]. The second study by Kimm-Brinson and Ramsdell [32] injected Medaka (*Oryzias latipes*) embryos with purified 0.1–8 ppm BTx-1. Upon hatching, morphologic abnormalities were commonly found in embryos exposed to doses of 1.0–4.0 ppm BTx. This included lateral curvature of the spinal column, as well as skull and eye malformations. Pronounced muscular activity after embryonic day 4 was also observed. Doses > 4.1 ppm produced embryos that developed but failed to hatch. These limited studies suggest *K. brevis* are toxic to larval fish and that large blooms have the potential to suppress larval recruitment.

Given the current lack of information and the implications for fisheries management, this study was undertaken to better quantify how exposure to different concentrations of *K. brevis* will impact larval fish survival. Laboratory spawned red porgy (*Pagrus pagrus*) larvae, whose range overlaps regions in the Gulf of Mexico where *K. brevis* blooms occur, were used as the representative larval fish species tested in these experiments. Specifically, larvae ages 4–26 days post hatch (dph), were incubated with different concentrations of the *K. brevis* strains NCMA2228, SP3, and SP1. The concentrations of cells employed were representative of cell concentrations found in low to high density blooms [1]. The *K. brevis* strains used were selected because each produces a distinctly different amount of BTxs, the primary suite of structurally related neurotoxins synthesized by this species.

## 2. Results

### 2.1. Effects of K. brevis on Larval Fish Survival

**Figure 1:**
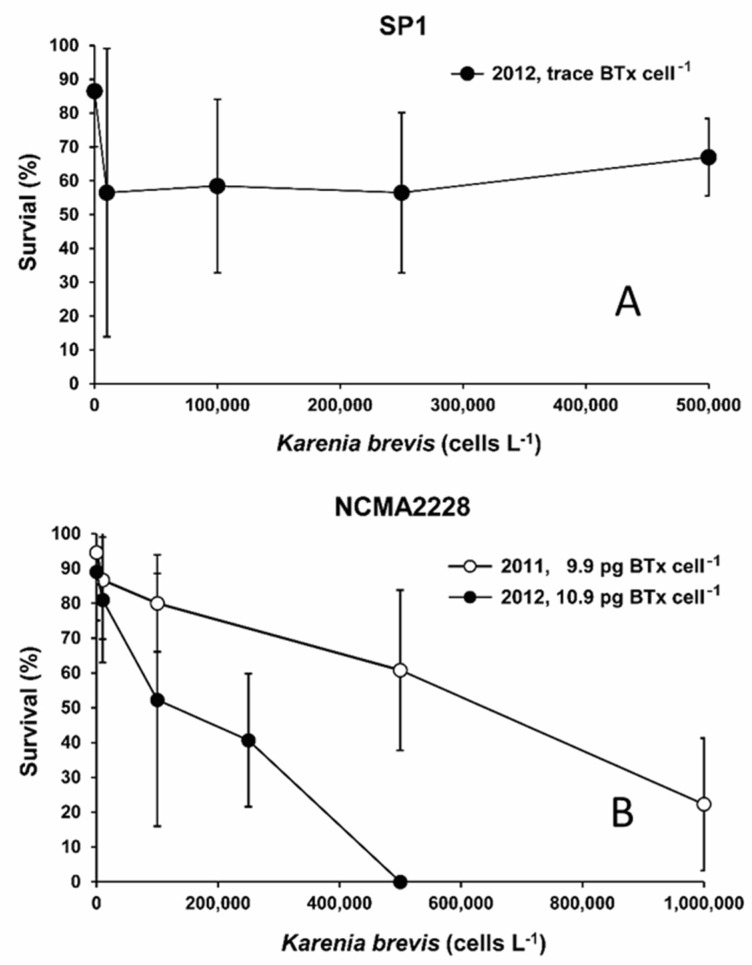

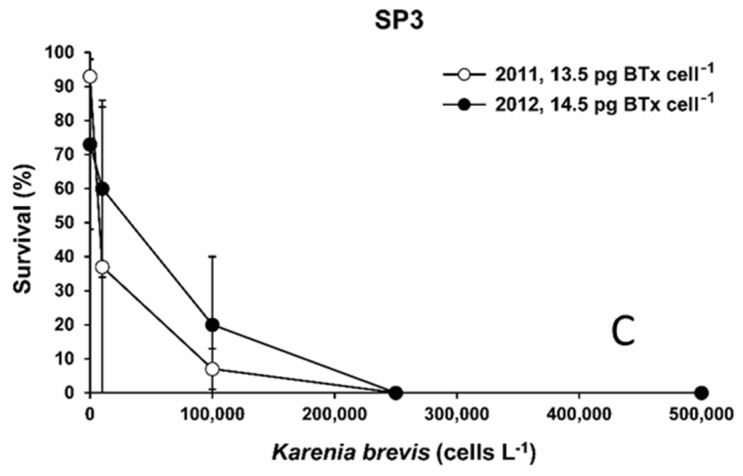
Red porgy larvae percent survival when exposed to the three *K. brevis* strains tested and produced either low, medium, or high concentrations of brevetoxin. Error bars are standard deviations of the mean. (**A**) Strain SP1 which contained trace amounts of pg BTx-1, 2, 3 cell^−1^. (**B**) Strain NCMA2228 averaged 9.9 pg BTx-1, 2, 3 cell^−1^ in 2011 and 10.9 pg BTx-1, 2, 3 cell^−1^ in 2012. (**C**) Strain SP3 averaged 13.5 pg BTx-1, 2, 3 cell^−1^ in 2011 and 14.5 pg BTx-1, 2, 3 cell^−1^ in 2012.

The data from individual triplicate exposure experiments of 6 to 21 dph larvae exposed to equivalent cell concentrations of either strain NCMA2228 or SP3 were also compared (Table S1). There was no evidence for any difference in the susceptibility of earlier versus later dph (non-yolk sac) larvae to the toxic effects of *K. brevis*. Consequently, the data for the different dph larvae exposed to a specific *K. brevis* strain in a given year were combined. The mean survival of red porgy larvae was found to be inversely proportional to BTx concentrations per cell in the three different *K. brevis* isolates tested (Figure 1). Exposure to SP1, which contains only trace amounts of BTx, caused an initial decline in survival that was the same regardless of the number of *K. brevis*. After the initial uniform drop, survivorship remained stable and did not decline as *K. brevis* cell concentrations increased (Figure 1A). NCMA2228 had an intermediate toxicity averaging 9.9 ± 2.2 pg BTx-1, -2, -3 cell^−1^ (*n* = 6) in the 2011 experiments and 10.9 ± 1.5 pg BTx-1, -2, -3 cell^−1^ (*n* = 3) in 2012 (Table S1). In both years survivorship declined in proportion to the number of cells present. The decline in survivorship versus cell concentration in 2012 was more pronounced when the BTx concentrations per cell were slightly higher (Figure 1B). The highest toxicity strain SP3, averaged 14.5 ± 1.3 pg BTx-1, -2, -3 cell^−1^ (*n* = 2) and caused an even more rapid decline in survivorship than exposure to either SP1 or NCMA2228 for an equivalent number of cells (Figure 1C). The only inter-year difference observed was the greater mortality of larvae in 2012 exposed to NCMA2228 compared to those exposed in 2011. A two-tailed *t*-test showed the difference between NCMA228 and SP3 at 100,000 cells for all replicates was not significant in 2012 (t_(16)_ = 0.18, ns), but highly significant in the 2011 experiments (t_(10)_ = 8.88, *p* < 0.001).

### 2.2. EC_50_ of Karenia brevis Versus Survivorship

**Figure 2:**
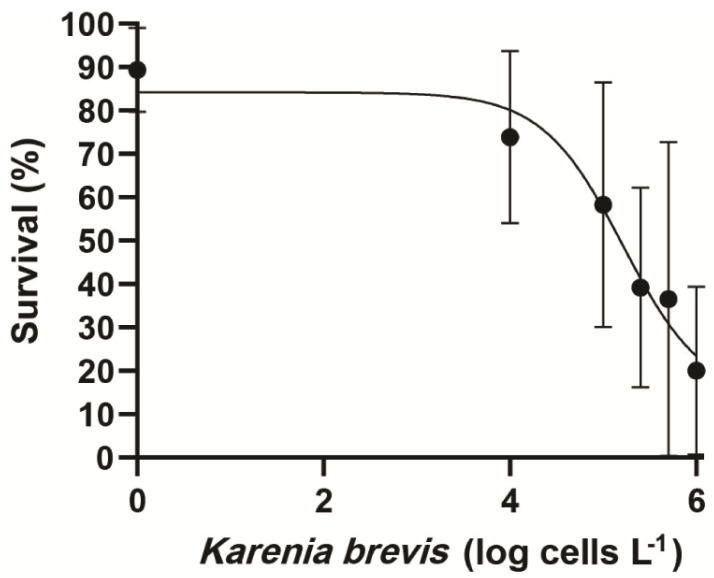
Dose-response curve created using combined data for all the 24 h experiments where red porgy larvae were exposed to varying cell concentrations of the three *K. brevis* strains tested. The black dots represent the mean survivorship for the different days post hatch larvae exposed to a given concentration of *Karenia brevis* ± 1 SD. The resulting estimated EC_50_ for the red porgy larvae was ~163,000 *K. brevis* cells L^−1^.

The estimated EC_50_ value was 163,000 *K. brevis* cells L^−1^ (Figure 2). The average toxicity for the *K. brevis* isolates used to calculate this EC_50_ value was 9.4 ± 4.8 pg BTx cell^−1^.

The corresponding estimated 24 h EC_50_ value based on the average BTx concentrations present in the different concentrations of whole cells tested was 17.8 pg L^−1^ (Figure S1). The data from the field indicate about 10% of the internal toxin pool is actually released into the water surrounding the cells [4].

### 2.3. Effect of Increased Exposure Time on Survivorship

**Figure 3:**
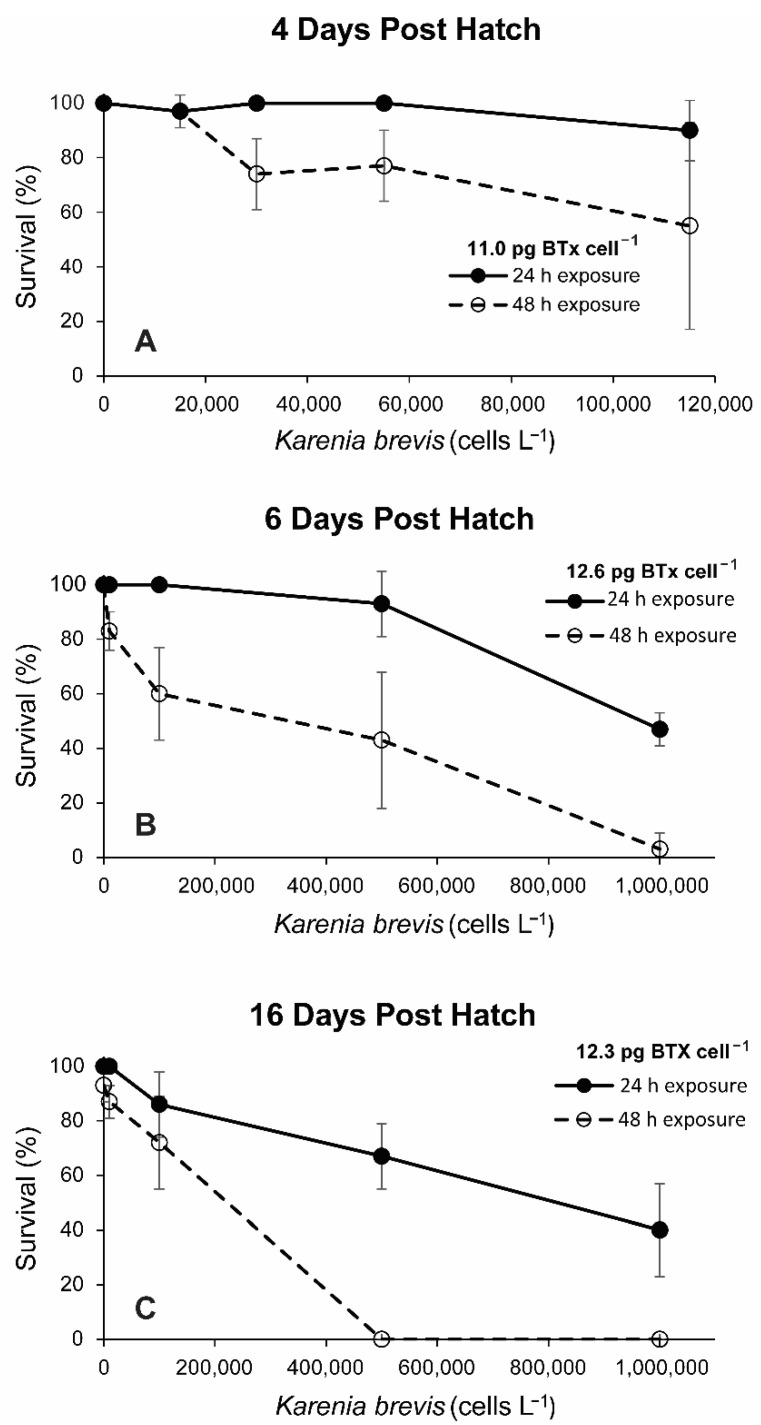
Difference in survivorship of red porgy larvae exposed to different concentrations of *K. brevis* strain NCMA2228 for either 24 or 48 h. (**A**) Exposure of four days post hatch larvae, (**B**) six days post hatch, and (**C**) sixteen days post hatch larvae.

Three trials were conducted where survivorship of the larvae exposed to NCMA2228 *K. brevis* were measured again at 48 h (Figure 3) to determine the extent to which increased exposure time caused elevated mortalities. The dph selected are representative of range in larval ages tested and include from 4 dph larvae which still retained their yolk sac and two later post yolk sac ages. The results consistently showed a significant decline between 24 and 48 h exposure times in the 4 dph (97% to 76%: t_(22)_ = 2.94, *p* = 0.008), 6 dph (85% to 47%: t_(22)_ = 3.72, *p* = 0.001), and 16 dph (e.g., 73% to 40%: t_(22)_ = 2.75, *p* = 0.011) larvae.

## 3. Discussion

*Karenia brevis* cell densities equivalent to those found in moderate to high density blooms were found to be highly toxic to red porgy larvae (*Pagrus pagrus*) (Figure 1 and Figure 2). Red porgy is a broadcast spawner which is widely distributed over the west Florida shelf, most commonly at depths between 10 to 80 m [33,34]. Larvae tend to migrate into the top 5 m of the water column where they can readily come into contact with *K. brevis* blooms. This species also exhibits a high degree of site fidelity and spawns from December through March. [35]. *K. brevis* blooms usually initiate in the late summer or early fall and typically persist over significant portions of the mid to inner west Florida shelf until mid to late-winter (January–February) [1,36]. Though less common, intense *K. brevis* blooms sometimes fail to decline in late winter and instead continue unabated through the spring and following summer [1]. *K. brevis* cells are highly mobile, often migrating up in the water column during the day and down at night. When upwelling conditions are favorable, *K. brevis* cells can also rapidly accumulate hydrodynamically over a few days to a week reaching concentrations far exceeding the EC_50_ values determined in this study [36,37]. Given their overleaping temporal and spatial distribution patterns, red porgy represent a good model species for evaluating the potential adverse impacts of *K. brevis* blooms on larval fish in the Gulf of Mexico.

The EC_50_ value of 163,000 *K. brevis* cells L^−1^ obtained in this study indicates the greatest red porgy larvae mortalities will occur at moderate to high bloom densities (100,000–10,000,000 cells L^−1^; Figure 2). This estimate is consistent with *K. brevis* cell densities (≥100,000 cells L^−1^) known to predictably kill diverse juvenile and adult fish species [1,30,38,39]. The corresponding EC_50_ value based on average BTx concentration in the cells was 17.8 pg L^−1^. Interestingly, shellfish larvae appear to require higher cellular concentrations of *Karenia*, in the range of 1,000,000 to 3,000,000 cells L^−1^, to induce significant mortality [40,41]. Though the EC_50_ value obtained in this study was markedly low compared to those obtained for shellfish larvae exposed to *K. brevis*, it may still represent an overestimate. This is because the cellular BTx levels for the three *K. brevis* strains used in this study averaged 9.4 ± 4.8 pg BTx cell^−1^ compared to the measured mean toxicity of *K. brevis* blooms in field of 18.2 ± 5.5 pg BTx cell^−1^ [4]. The cause of these toxicity difference is primarily due to our experiments being conducted using exponentially growing cells. These fast-growing, non-nutrient limited cells exhibit lower toxicities than the slower growing, nutrient limited cells often encountered in the field [4,42]. For example, NCMA2228 averages 9 pg BTx-1, -2, -3 cell^−1^ in exponential phase growth, 19.2 pg BTx-1, -2, -3 cell^−1^ under nitrate limitation and 42.7 pg BTx-1,2,3 cell^−1^ under phosphate limitation [43,44]. Similarly, SP3 averages 13 pg BTx-1, -2, -3 cell-1 when in exponential nutrient replete growth and 19.7 when nitrogen limited [43]. Consequently, had field *K. brevis* populations containing almost two-fold higher cellular BTx concentrations been used instead of the strains employed in this study, a lower EC_50_ estimate would be predicted. If true, exposure to even relatively low-density *K. brevis* blooms (50,000 cells L^−1^ or less) may cause increased larval mortalities.

Results from this study also showed that as exposure time was increased from 24 to 48 h, red porgy percent mortalities increased substantially regardless of larval age (Figure 3). This indicates the longer the larvae are exposed to relatively low *K. brevis* cell concentrations in the field, the greater the resultant larval mortalities. Riley et al. [31] noted 21 dph red drum larvae needed longer exposure times to reach the same level of mortality compared to yolk-sac larvae. The data on *K. brevis* cell concentration versus mortality data for the 6–21 post yolk-sac larvae indicated each age was equally susceptible to the toxic effects of *K. brevis* (Table S1). The data for the 4 dph yolk-sac larvae, in contrast, were insufficient to determine if these larvae were more or less susceptible to *K. brevis* toxicity compared to the post yolk-sac larvae.

Given the high toxicity of *K. brevis* and its wide distribution along the west Florida shelf, it is likely this toxic dinoflagellate may periodically cause larval mortalities sufficient to reduce inter-annual recruitment and year-class strength of important fish stocks. Any adverse population and community level associated effects would be compounded by the concomitant increases in juvenile and adult fish mortalities associated with *K. brevis* blooms [30,45,46,47]. Further support for the potential negative impacts on fish stocks comes from the reduction in bivalve larvae recruitment [39,48,49,50] and mass mortality of benthic organisms including corals observed during bloom during *K. brevis* blooms [22,45,51,52,53,54]. In the latter case, radical shifts in the benthic community structure persisting for over two years following a major bloom have been observed. Any significant reductions in year-class strength are of particular concern for already overfished species in the Gulf of Mexico. Cumulatively, these data suggest more detailed correlative studies examining decadal changes in the nekton community and year-class strength of fish species following moderate to severe *K. brevis* bloom years are needed to fully elucidate the effects of bloom on the resilience of fisheries stocks in this region [46,55].

Though the toxicity of the *K. brevis* isolates correlated with their ability to produce different levels of BTx, it is important to note no experiments were conducted using purified BTx to conclusively demonstrate these neurotoxins per se were definitively responsible for the observed mortalities. Early histopathological studies indicated many fish exposed to *K. brevis* died from chronic tissue damage rather than neuro-intoxication [38]. One of the most likely candidates for such damage would be the production of reactive oxygen species (ROS) superoxide and hydrogen peroxide, which is known to be the mechanism of toxicity for some harmful algal bloom species [56]. *Karenia brevis* cells are known to produce low levels of intracellular ROS which are reported to both increase in concentration with culture age and fluctuated in a phasing diel pattern [57]. Other work confirmed *K. brevis* does indeed produce intermediate levels of extracellular ROS, but in contrast showed the production varied inversely with cell density [58]. The later study also failed to detect active ROS in spent media of *K. brevis* cultures. Recent surveys, while documenting the importance of ROS toxicity in *K. mikimotoi*, did not identify ROS as a major mechanism by which *K. brevis* causes toxicity [56,59]. Cumulatively, these data indicate the ROS is unlikely to account for the elevated larval mortality observed with increasing *K. brevis* concentrations.

Instead, *K. brevis* toxicity is widely attributed to BTXs and their derivatives being neurotoxic, hemolytic, and potent inducers of oxidative stress [60,61,62,63,64,65]. Its specific mode of action in blocking voltage gated sodium channels of most organisms in particular [28,66], suggests BTxs as the most parsimonious explanation for the observed toxicity of *K. brevis* to larval red porgy [60]. Regardless of the specific toxins involved, the results of this study indicate moderate to intense blooms of *K. brevis* may cause larval fish mortalities sufficient to affect population dynamics of ecologically and commercially important fish stocks in the Gulf of Mexico.

## 4. Materials and Methods

### 4.1. Larval Fish Rearing

Experiments were conducted using red porgy (*Pagrus pagrus*) larvae reared from two volitional spawns of brood stock held at the NOAA Beaufort Laboratory, Beaufort, NC, USA; one in 2011 and one in 2012. Viable eggs were separated by placing them in a 1 L graduated cylinder and pouring off the viable eggs into a 100 L hatching cone kept at 19 °C by a ¾ horsepower heater/chiller. The larvae hatched 48 h later and were concentrated into a smaller cone via a siphon. Larvae were placed in 100 L recirculating (400% daily exchange) tanks at 23 °C and salinity of 34 [67]. Photoperiod was maintained at 16:8 light:dark cycle (07:00–23:00) from a single 90-watt Sylvania daylight spectrum fluorescent bulb. Aeration was supplied via a single flexible air diffuser around the center of a 500 µm mesh screen overflow. Larvae were fed S-type rotifers (*Brachionus rotundiformis*) enriched with Rotigrow plus (Reed Mariculture; Campbell, CA USA) starting 2 days post hatch (dph) at a density of 3 rotifers mL^−1^ and increased daily until 12 rotifers mL^−1^ was reached. Feeding took place once per day (08:00) by hand and an additional three times per day (12:00, 16:00, and 20:00) with refrigerated (10 °C) feed pumped into the tanks via a peristaltic pump controlled by a timer. A non-viable, condensed algae paste (Nanno, Reed Mariculture; Campbell, CA USA) was added twice daily at a concentration of 750,000 cells mL^−1^ starting at 1 dph as background. Instar I stage brine shrimp (*Artemia salina*) were co-fed with rotifers starting 15 dph and until 20 dph when Instar II stage enriched *Artemia* (DC DHA SeECo, INVE, Salt Lake City, UT, USA) were fed exclusively. *Artemia* were enriched 24 h prior to feeding as prescribed by the manufacturer’s protocols. *Artemia* (1–3 *Artemia* mL^−1^) were fed the same way as the rotifers. Otohime C1 Fish Diet (Reed Mariculture; Campbell, CA, USA) was fed starting at 15 dph in addition to live organisms.

Red porgy (Sparidae; *Pagrus pagrus*) was selected as a representative species that exhibits an epipelagic larval stage with high likelihood of natural exposure to BTx in coastal marine waters [35]. Red porgy exhibit a two-phase life cycle where more sedentary and demersal adults produce pelagic larvae that are widely dispersive by hydrodynamic forces such as wind, tide, and turbulence [67]. By comparing larvae hatched in 2011 versus 2012, it was possible to evaluate if inter-year differences in the reared larvae affected susceptibility to *K. brevis* toxicity. The range of *K. brevis* concentrations tested represent bloom density levels from background to moderate as defined by the Florida Fish and Wildlife Research Institute [1]. The larval ages tested coincided with key developmental stages including the development of mouthparts and gills.

### 4.2. Karenia brevis Cultures

Three *K. brevis* strains, NCMA2228, SP3, and SP1, were used in this study. NCMA2228 was obtained from the National Center for Marine Algae and Microbiota and was isolated from Mote Marine Laboratory New Pass Dock, Sarasota, FL, USA. Strains SP1 and SP3 were provided by Dr. Edward Buskey of the University of Texas Marine Science Institute, Port Aransas TX, USA. Both SP1 and SP3 were isolated during a bloom event off the coast of Brownsville, Texas in October 1999 by S. Pargee, University of Texas Marine Science Institute, Port Aransas. *K. brevis* isolates have long been known to vary in toxicity [68] and these strains were selected because they are known to contain either trace (SP1), intermediate (NCMA2228), or high (SP3) intercellular levels of BTx [43,68,69].

*Karenia brevis* cells were grown in 2.5 L semi-continuous batch cultures as described in Hardison et al. [41]. Cells were cultured in a Percival Scientific (Perry, IA, USA) model I-36VLX incubator maintained at a constant temperature of 23 °C and on a 14 h:10 h daily light:dark cycle. Photosynthetically active radiation (PAR) was provided at an intensity of 120 µmol quanta m^−2^ s^−1^ via vertically mounted fluorescent Duro-test Vita-lites. PAR intensity was measured with a Biospherical Instruments Inc. (San Diego, CA, USA) QSL-100 4π wand type light meter.

Media consisted of 1.0 L of 0.2 µm filtered Gulf Stream seawater (salinity 36) held in 2.5 L polycarbonate bottles. The media contained added vitamins (0.074 nM vitamin B12, 0.4 nM biotin, and 60 nM thiamine), 10 nM Na2SeO3, and an EDTA trace metal buffer system (100 mM EDTA, 1 mM FeEDTA, 50 nM MnCl_2_, 40 nM CuCl_2_, 100 nM ZnSO_4_, and 40 nM CoCl_2_). Nutrient-replete culture media contained 64 µM NaNO_3_ and 4 μM NaH_2_PO_4_ (N:P = 16:1, the Redfield ratio). Media were sterilized by microwave treatment [41]. Culture pH was measured initially and throughout each experiment with a Thermo Scientific (Waltham, MA, USA) Orion 3 Star pH meter equipped with a Ross ultra-combination pH electrode to ensure no carbon dioxide limitation occurred. Culture pH ranged from 8.10 to 8.30.

Cell concentrations were measured with a Beckman Coulter Inc. (Brea, CA, USA) Multisizer 3 electronic particle counter (0.5-mL sample volume) equipped with a 100-µm aperture. Cell growth curves were constructed as semi-log plots of total biovolume (μLcells Lmedia^−1^) versus time in days. Total biovolume (μLcells Lmedia^−1^) was calculated by multiplying the cellular concentration (cells L^−1^) by the mean volume per cell (µm^3^ cell^−1^). Specific growth rates were computed from linear regressions of the natural log of total cell biovolume versus time after correcting for culture dilution [70]. Only exponentially growing, nutrient sufficient *K. brevis* cells were harvested and used in the exposure studies. Specific growth rates for NCMA2228, SP3, and SP1 were 0.45 d^−1^, 0.37 d^−1^, and 0.31 d^−1^, respectively.

### 4.3. Brevetoxin Extractions and Quantification

Each time cells were used in an exposure experiment, aliquots of cell cultures were mixed 1:1 by volume with ethyl acetate for brevetoxin (BTx) determination using the methods described in Hardison et al. [43]. The ethyl acetate mixtures were sonicated for three minutes with a Branson Sonifier 250 (Branson Ultrasonics Corporation, Danbury, CT, USA) and complete cell disruption was confirmed by microscopy. The aliquots were desalted with Milli-Q water (Millipore Sigma, Burlington, MA, USA) and concentrated with a rotovap. Extraction efficiency was determined in each aliquot by the addition of an internal standard BTx-42-acetate (kindly provided by Dr. Dan Baden, University of North Carolina at Wilmington, Wilmington, NC, USA) and typically ranged from 95–100% (Lekan and Tomas, 2010). Brevetoxin concentrations in each aliquot were measured using an Agilent 1100 LC (Agilent Technologies, Santa Clara, CA, USA) coupled to a Thermo Finnigan TSQ Quantum triple quadrupole mass spectrometer (Thermo Fisher Scientific, Waltham, MA, USA), with an electrospray ion source interface. The LC-MS-MS methodology used has been described in detail [71,72]. An external standard curve of three purified brevetoxins (BTx-1, -2, -3) (World Ocean Solutions, Wilmington, NC, USA) was used to quantify the amounts of BTx-1, -2, -3 in each of the aliquots for each trial. Brevetoxin levels in this study are the mean total brevetoxin levels (pg cell^−1^), which were determined by taking an average of the sum BTx-1, -2, -3 across six aliquots in each trial. The main reason for measuring toxicity each time cells were used is that toxicity can vary even when grown under constant conditions [64].

### 4.4. Exposure Experiments

Experimental systems consisted of 1 L Pyrex beakers placed on the laboratory bench at room temperature (21 °C), full strength filtered seawater (35), and 24 h overhead fluorescent lighting. Experimental controls contained filtered seawater, larvae, and age-appropriate food, while experimental treatments contained filtered seawater, larvae, age-appropriate food source, and *K. brevis*. Three beakers (triplicates) of seven to ten larvae were used in each control and treatment. In 2011, larvae were initially tested at 4 dph using *K. brevis* strain NCMA2228 at concentrations from 0–115,000 cells L^−1^; however, survival was high across treatments, so a higher range of cell concentrations was tested in the remaining trials. Subsequently, larvae were exposed to NCMA2228 at 6, 8, 14, 16, and 21 dph at *K. brevis* cell concentrations from 0–500,000 or 0–1,000,000 cells L^−1^ depending on the toxicity of the *K. brevis* strain being employed. Similarly, 13 dph larvae were exposed to *K. brevis* strain SP3 at concentrations from 0–1,000,000 cells L^−1^. In 2012, preliminary trials with 5 dph larvae indicated the larvae were more sensitive to the slightly more toxic *K. brevis* strains used, when compared to the 2011 experiments. In this case, a lower range of cell concentrations were tested. Specifically, 7, 11, and 26 dph were exposed to *K. brevis* strain NCMA2228, 11 dph larvae to strain SP3, and 18 and 21 dph to strain SP1 at concentrations ranging from 0–500,000 cells L^−1^. Cell concentrations were maintained throughout the duration of each trial and care was taken to prevent *K. brevis* cell damage and inadvertent release of excess toxin, which was confirmed by microscopically examining the cells to ensure they were healthy and intact. The average cell concentration for each of the three *K. brevis* isolates were then plotted versus percent survival ± one standard deviation and the toxicity of the isolate for each experiment noted.

### 4.5. Estimating the EC_50_ Value

The 24 h-EC_50_ value for larval red porgy exposed to different concentrations of *K. brevis* was calculated using the Graphpad Prism^®^ (GraphPad Software, Inc., La Jolla, CA, USA) program. Input data were the *K. brevis* cell numbers and the corresponding average 24 h survivorship ± one standard deviation for all of the experiments. Support for combining the data from multiple isolates having different BTx levels to estimate an average EC_50_ value comes from a field study by Tester et al. [4]. They found an average toxicity of 18.2 ± 5.5 pg BTx per *K. brevis* cell^−1^ during a bloom event off the west coast of Florida. The measured toxicity of the *K. brevis* isolates used for each experimental treatment in this study was averaged to determine how closely their mean toxicity matched BTx concentrations per cell measured in the field. It should be noted that in addition to simulating average field toxicity, combining larval survivorship data for strains producing different amounts of BTX also increased the observed variance in percent survivorship for each *K. brevis* cell concentration tested.

An 24 h-EC_50_ value for larval red porgy exposure based on pg BTx L^−1^ was also calculated as above using the same percent survivorship data. The only difference was that mean toxin content for each of the cell concentrations variously tested in the 24-h studies (0, 10,000, 100,000, 250,000, 500,000, or 1,000,000 cells L^−1^) was used instead of cell concertation as input to the Graphpad Prism program. The BTX values used to calculate the mean toxin values for each concentration of *K. brevis* cells tested were obtained by multiplying the measured BTx concentration (pg cell^−1^) for the strain used in each individual experiment by the specific concentration of *Karenia* cells L^−1^ being tested. The BTx concentrations were then sorted into groups corresponding to the cell concentration tested and corresponding average BTx concentrations calculated.

### 4.6. Examination of How Prolonged Exposure to K. brevis Impacts Survivorship of Red Porgy Larvae

For a subset of the experiments using *K. brevis* strain NCMA2228, the incubations with 4, 6, and 16 dph larvae from 2011 were allowed to proceed for 48 h and the resulting mortalities recorded (Figure 1). The toxicity of the isolates employed in these experiments were similar ranging from 11 to 12.3 pg BTx cell^−1^. This provided a consistency in *K. brevis* concentration that allowed an estimate of how survivorship might be affected by exposure to toxic *K. brevis* blooms over longer periods of time. Statistical differences in mean survivorship between the 24 and 48 h exposure treatments was determined using a *t*-test.

## Data Availability

Not applicable. All data are shown in the manuscript or in supplementary Table S1.

## Supplementary Materials

The following supporting information can be downloaded at: https://www.mdpi.com/article/10.3390/toxins14070439/s1, Table S1, Summary data for each of the 24 h and 48 h experiments carried out in this study where red porgy larvae were variously exposed to three different Karenia brevis isolates. Figure S1: Dose-response curve created using combined data for all the 24 h experiments where red porgy larvae were exposed to concentrations of the three K. brevis strains which contained different internal BTx concentrations (Table S1). The average BTx concentration for each concentration of K. brevis cells testes were calculated and used in conjunction with their corresponding mean survival rates (±SD) to estimate a BTx-based EC50. The resulting EC50 value was ~17.8 pg L^-1^ in whole *K. brevis* cells. Field studies indicated about 10% of the internal BTx concentrations are released into the water column [4].
