# Supplementary material for: The Effects of the Harmful Algal Bloom Species Karenia brevis on Survival of Red Porgy (Pagrus pagrus) Larvae
Source: Toxins (Basel). 2022 Jun 28;14(7):439. doi: 10.3390/toxins14070439 (PMC9317425; doi:10.3390/toxins14070439)
Supplement: Supplementary file 1 [file toxins-14-00439-s001.zip › toxins-1755868-supplementary.pdf]

The Effects of the Harmful Algal Bloom Species *Karenia brevis* on Survival of Red Porgy (*Pagrus pagrus*) Larvae

Richard Wayne Litaker, Alex K. Bogdanoff, Donnie Ransom Hardison, William C. Holland, Andrew Ostrowski and James A. Morris

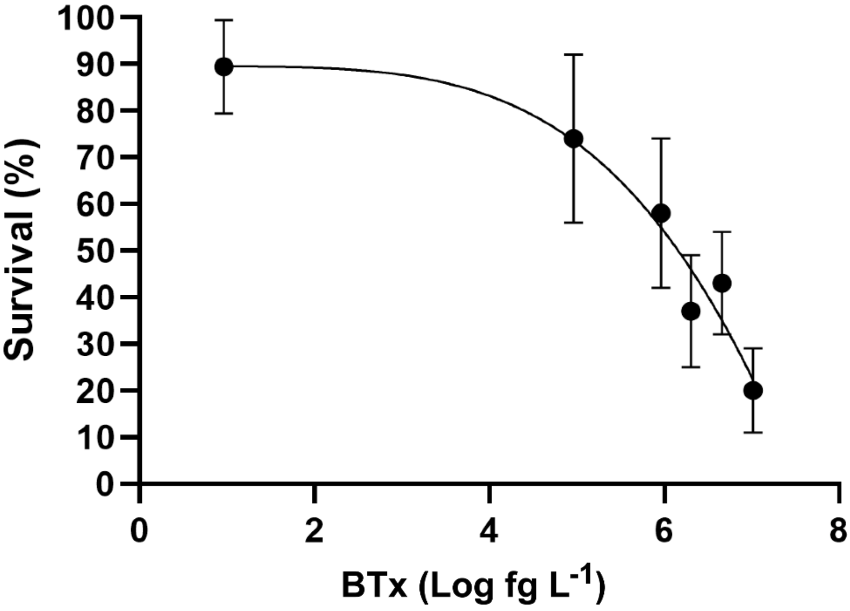

Figure S1. Dose-response curve created using combined data for all the 24 h experiments where red porgy larvae were exposed to concentrations of the three K. brevis strains which contained different internal BTx concentrations (Table S1). The average BTx concentration for each concentration of K. brevis cells testes were calculated and used in conjunction with their corresponding mean survival rates (SD) to estimate a BTx-based EC50 . That value was ~17.8 pg L-1 in whole K. brevis cells. Field studies indicated about 10% of the internal BTx concentrations are released into the water column [4].

Table S1. Summary data for each of the 24h and 48h experiments carried out in this study where red porgy larvae were variously exposed to three different *Karenia brevis* isolates.

Date & Strain	Larval age days post hatch (dph) & Mean cellular brevetoxin concentrations (BTxs 1-2-3)	cells L^{-1}	Number of larvae	24-hr % survival (mean ± SD)	48-hr % survival (mean ± SD)
2011 NCMA2228	4 dph 11.0 pg $cell^{-1}$	0	28	100 ± 0	100 ± 0
		15,000	28	97 ± 6	97 ± 6
		30,000	28	100 ± 0	74 ± 13
		55,000	27	100 ± 0	77 ± 13
		115,000	30	90 ± 11	55 ± 38
	6 dph 12.6 pg $cell^{-1}$	0	29	100 ± 0	100 ± 0
		10,000	28	100 ± 0	83 ± 7
		100,000	30	100 ± 0	60 ± 17
		500,000	30	93 ± 12	43 ± 25
		1,000,000	30	47 ± 6	3 ± 6
	8 dph 6.7 pg $cell^{-1}$	0	30	87 ± 6	-
		10,000	30	67 ± 12	
		100,000	30	67 ± 6	
		500,000	30	30 ± 10	
		1,000,000	30	3 ± 6	
	14 dph 9.9 pg $cell^{-1}$	0	30	97 ± 6	-
		10,000	30	93 ± 6	
		100,000	30	80 ± 10	
		500,000	30	63 ± 29	
		1,000,000	30	17 ± 21	
	16 dph 12.3 pg $cell^{-1}$	0	27	100 ± 0	93 ± 6
		10,000	30	100 ± 0	87 ± 6
		100,000	29	86 ± 12	72 ± 17
		500,000	30	67 ± 12	0 ± 0
		1,000,000	30	40 ± 17	0 ± 0
	21 dph 6.9 pg $cell^{-1}$	0	24	83 ± 7	-
		10,000	24	67 ± 7	
		100,000	24	67 ± 7	
		500,000	24	51 ± 20	
		1,000,000	24	13 ± 13	
2011 SP3	13 dph 13.5 pg $cell^{-1}$	0	29	93 ± 12	-
		10,000	30	37 ± 47	
		100,000	30	7 ± 6	
		500,000	29	0 ± 0	
		1,000,000	30	0 ± 0	
2012 NCMA2228	7 dph 11.4 pg $cell^{-1}$	0	30	73 ± 21	-
		10,000	30	77 ± 6	
		100,000	30	63 ± 12	
		250,000	29	24 ± 21	
		500,000	30	0 ± 0	
	11 dph 12.1 pg $cell^{-1}$	0	30	97 ± 6	-
		10,000	30	90 ± 10	

		100,000	30	30 ± 20	
		250,000	29	48 ± 3	
		500,000	30	0 ± 0	
	26 dph<br>9.2 pg cell^{-1}	0	30	97 ± 6	-
		10,000	30	83 ± 12	
		100,000	30	63± 47	
		250,000	30	50 ± 0	
		500,000	30	0 ± 0	
2012 SP3	11 dph<br>15.4 pg cell^{-1}	0	30	73 ± 25	-
		10,000	30	60 ± 26	
		100,000	30	20 ± 20	
		250,000	30	0 ± 0	
		500,000	30	0 ± 0	
2012 SP1	18 dph<br>trace	0	30	90 ± 10	-
		10,000	30	60 ± 40	
		100,000	30	40 ± 17	
		250,000	30	50 ± 30	
		500,000	30	87 ± 6	
	21 dph<br>trace	0	30	83 ± 21	-
		10,000	30	53 ± 45	
		100,000	30	77 ± 32	
		250,000	30	47 ± 15	
		500,000	30	63 ± 15	
